# Evidence for the transition from primary to peritectic phase growth during solidification of undercooled Ni-Zr alloy levitated by electromagnetic field

**DOI:** 10.1038/srep39042

**Published:** 2016-12-13

**Authors:** P. Lü, K. Zhou, H. P. Wang

**Affiliations:** 1 MOE Key Laboratory of Space Applied Physics and Chemistry, Department of Applied Physics, Northwestern Polytechnical University, Xi’an 710072, P. R. China

## Abstract

The Ni_83.25_Zr_16.75_ peritectic alloy was undercooled by electromagnetic levitation method up to 198 K. The measured dendritic growth velocity shows a steep acceleration at a critical undercooling of Δ*T*_crit_ = 124 K, which provides an evidence of the transition of the primary growth mode from Ni_7_Zr_2_ phase to peritectic phase Ni_5_Zr. This is ascertained by combining the temperature-time profile and the evolution of the solidified microstructures. Below the critical undercooling, the solidified microstructure is composed of coarse Ni_7_Zr_2_ dendrites, peritectic phase Ni_5_Zr and eutectic structure. However, beyond the critical undercooling, only a small amount of Ni_7_Zr_2_ phase appears in the solidified microstructure. The dendritic growth mechanism of Ni_7_Zr_2_ phase is mainly governed by solute diffusion. While, the dendritic growth mechanism of Ni_5_Zr phase is mainly controlled by thermal diffusion and liquid-solid interface atomic attachment kinetics.

Peritectic solidification is frequently encountered among metallic alloy systems, such as Fe-Ni, Fe-Al, Al-Ni, Ni-Zr, etc.^1^,^2^,^3^,^4^,^5^, which is of great importance in preparing various commercial component materials. Recently, a transition of the primary growth mode from primary phase to peritectic phase during the solidification of undercooled peritectic alloys has been paid considerable attention^6^,^7^,^8^,^9^,^10^. The underlying reason is that the transition of the primary growth mode may result in the formation of phase-pure peritectic phase in the final solidified microstructure, and thus improve the performance of peritectic alloys^7^. Tourret *et al*.^4^,^5^ investigated the multiple phase transformations of Al-Ni peritectic alloys by electromagnetic levitation and gas atomization methods, and found that peritectic phase Al_3_Ni preferentially grows when the droplet diameter is 10 μm for gas atomized Ni-80 at% Al hyperperitectic alloy, which is attributed to the competition between the cooling kinetics and the diffusion kinetics. Phanikumar *et al*.^11^ found that once the undercooling (unless stated otherwise, any mention of undercooling in the paper refers to nucleation undercooling) exceeds a critical value of about 110 K, the solidified microstructure consists of only peritectic phase in Fe-25%Ge peritectic alloy processed by an electromagnetic levitator. Leonhardt *et al*.^12^ reported that a transition of the primary growth mode from primary bcc-Mo to peritectic σ-phase was revealed if the undercooling of Fe_47_Mo_53_ alloy is beyond 345 K. However, although some significant progresses have been reported, direct experimental evidence of the transition of the primary growth mode from the primary phase to peritectic phase is rather limited. Previous studies revealed this transition of primary growth mode mainly by the evolution of solidified microstructures. Actually, dendritic growth is the major growth mode in undercooled melts, the velocity of which can provide valuable insight into the transition of primary growth mode. Nevertheless, few investigations of dendritic growth kinetics in undercooled peritectic alloys have been reported.

As a typical peritectic alloy system, Ni-Zr binary alloy system has aroused particular interests due to its good glass forming ability in a wide compositional range^13^,^14^,^15^ as well as its abundant intermetallic compounds^16^,^17^,^18^. Ni_83.25_Zr_16.75_ is a peritectic composition in Ni-Zr alloy system, whose primary phase and peritectic phase are both intermetallic compounds. The solidification behavior for this type of peritectic alloy is complicated but also considerably important for deep and comprehensive understanding of peritectic solidification under highly undercooled condition. Therefore, the objective of this work is to investigate the transition of the primary growth mode from Ni_7_Zr_2_ phase to peritectic phase Ni_5_Zr during the solidification of undercooled Ni_83.25_Zr_16.75_ peritectic alloy by the measured dendritic growth velocity and solidified microstructures. Meanwhile, the dendritic growth kinetics of Ni_7_Zr_2_ phase and Ni_5_Zr phase is also studied to reveal the evolution of solidified microstructure.

## Results and Discussion

**Figure 1:**
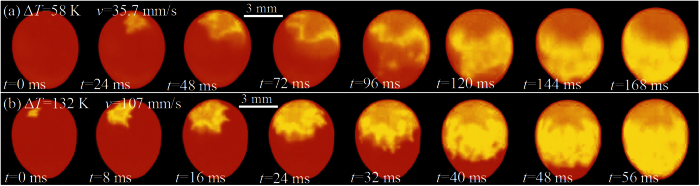
High-speed camera images of recalescence front of undercooled Ni_83.25_Zr_16.75_ peritectic samples at different undercoolings. The red part is the undercooled liquid, and the yellow part is the recalescing solid. The time interval between two adjacent images is 24 ms and 8 ms, respectively. (**a**) Δ*T* = 58 K; (**b**) Δ*T* = 132 K.

The high-speed camera technique can allow the visualization of the propagation front, which is a feasible approach to investigate the rapid solidification process. Figure 1 shows a few snapshots of rapid solidification front in undercooled Ni_83.25_Zr_16.75_ peritectic melts at different undercoolings, which were captured by a Red-lake HG 100 K high-speed camera with the resolution of 24 bits (color) pixel depth. The yellow area corresponds to the solid due to the released heat, and the red area corresponds to the undercooled liquid. Noticeably, solidification starts at the upper surface of the sample and proceeds to the lower part. The propagating front appears ambiguous for low undercooling, and gives distinct feature of dendritic structure for high undercooling. The dendritic growth velocity can be determined from the sequence of projected images captured by the high-speed camera, which is coincident with the value measured by a photoelectric detector.

**Figure 2:**
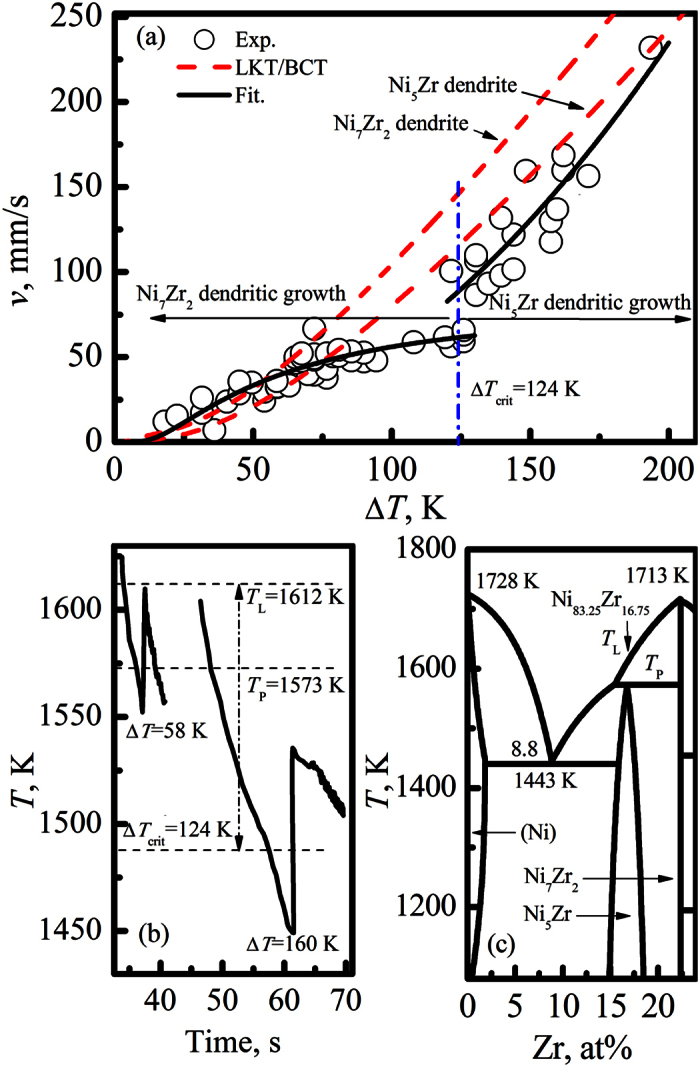
(**a**) Dendritic growth velocity versus undercooling. (**b**) Temperature-time recalescence characteristics at different undercoolings. (**c**) The left part of Ni-Zr binary phase diagram^24^.

The results of dendritic growth velocity with different undercoolings in Ni_83.25_Zr_16.75_ melts are presented in Fig. 2(a), which was measured by a photoelectric detector. The maximum undercooling obtained in the present work is about 198 K. It is obvious that the measured dendritic growth velocity continuously increases with the enhancement of the undercooling. When the undercooling is smaller than a critical value of Δ*T*_crit_ = 124 K, the growth velocity appears sluggishly. Once the undercooling exceeds the critical value of Δ*T*_crit_ = 124 K, the growth velocity increases rapidly. More importantly, a steep rise of the growth velocity is observed at the critical undercooling of Δ*T*_crit_ = 124 K, which jumps from 61 mm/s to 88 mm/s. Such a phenomenon implies that a transition of the primary growth mode from Ni_7_Zr_2_ phase to peritectic phase Ni_5_Zr occurs. The equilibrium solidification of Ni_7_Zr_2_ phase is replaced by the direct growth of peritectic phase Ni_5_Zr if the undercooling exceeds the critical value of Δ*T*_crit_ = 124 K. As for Ni_83.25_Zr_16.75_ peritectic alloy, the liquidus temperature of Ni_7_Zr_2_ phase is just higher than that of Ni_5_Zr phase by about 39 K. If the undercooling of the melt goes through the peritectic temperature *T*_p_, the peritectic phase Ni_5_Zr becomes a metastable phase and may form directly from the undercooled melt despite the low thermodynamic driving force. Our previous study^3^ has revealed that the peritectic phase Ni_5_Zr can form directly from the undercooled melt by completely suppressing the growth of Ni_7_Zr_2_ phase if the droplet diameter is less than a critical value in the drop tube experiments.

To verify the transition of the primary growth mode, the temperature-time curves during undercooling and rapid solidification of Ni_83.25_Zr_16.75_ peritectic melts at two different undercoolings are illustrated in Fig. 2(b). The recalescence behavior is characterized by a steep temperature rise detected by the pyrometer. For low undercooling of 58 K, the temperature of the melt after recalescence rises nearly to the liquidus temperature *T*_L_, which gives an indication of growth of primary Ni_7_Zr_2_ dendrites. However, for high undercooling of 160 K, the recalescence process is observed to stop below the peritectic temperature *T*_P_. This may give an evidence for a change of growth mode that the growth of Ni_7_Zr_2_ phase is replaced by the growth of peritectic phase Ni_5_Zr if the undercooling exceeds the critical value of 124 K.

**Figure 3:**
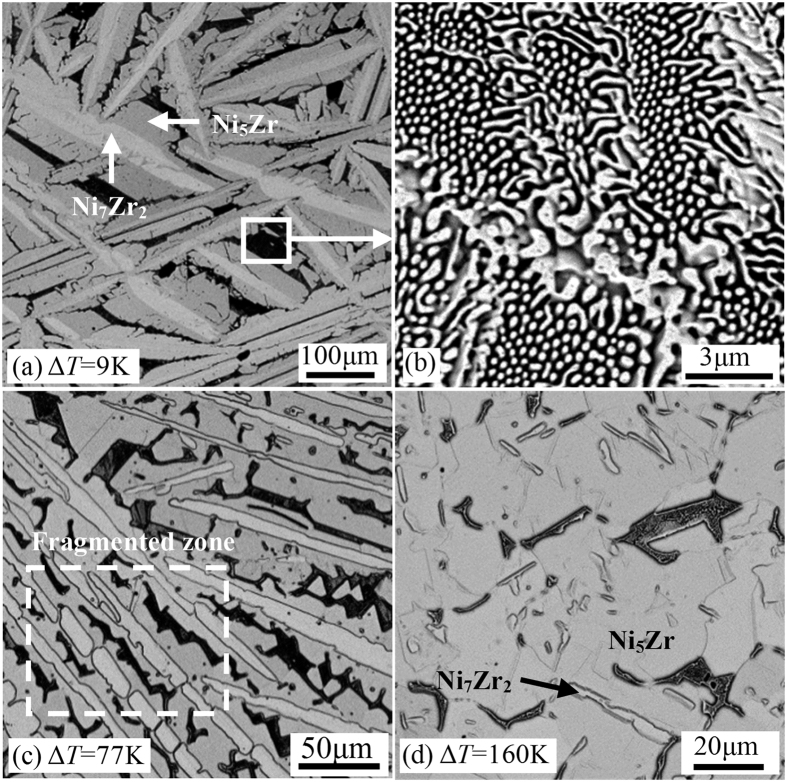
Solidified microstructures of undercooled Ni_83.25_Zr_16.75_ peritectic alloy at undercooling of (**a**,**b**) Δ*T* = 9 K, (**c**) Δ*T* = 77 K, and (d) Δ*T* = 160 K.

The microstructures of Ni_83.25_Zr_16.75_ peritectic samples solidified at different undercoolings are shown in Fig. 3, in which both the Ni_7_Zr_2_ phase and Ni_5_Zr phase have been marked. The solidified microstructure consists of Ni_7_Zr_2_ dendrites, peritectic phase Ni_5_Zr and inter-dendritic eutectic microstructure for low undercooling of Δ*T* = 9 K, as illustrated in Fig. 3(a). Apparently, the Ni_7_Zr_2_ phase exhibits coarse and developed dendrites, which is enwrapped by peritectic phase Ni_5_Zr. Figure 3(b) is an enlarged view of the inter-dendritic eutectic microstructure. It can be seen that the morphology is characterized by rod-like eutectic structure, which is the mixture of (Ni) and Ni_5_Zr phases. With the enhancement of undercooling, the fragment of Ni_7_Zr_2_ dendrites occurs which seems like the primary Ni_7_Zr_2_ dendrite trunks have partially been transformed to peritectic phase, and the fragmented zone is marked as a box, as shown in Fig. 3(c). However, when the undercooling is beyond the critical value of Δ*T*_crit_ = 124 K, a significant change of microstructure takes place. The solidified microstructure for a high undercooling of Δ*T* = 160 K is composed of a small amount of Ni_7_Zr_2_ phase, predominant Ni_5_Zr phase and eutectic microstructure, as presented in Fig. 3(d). It is evident that the amount of Ni_7_Zr_2_ phase is very low, and the Ni_7_Zr_2_ phase seems to be decomposed and nearly disappears.

**Figure 4:**
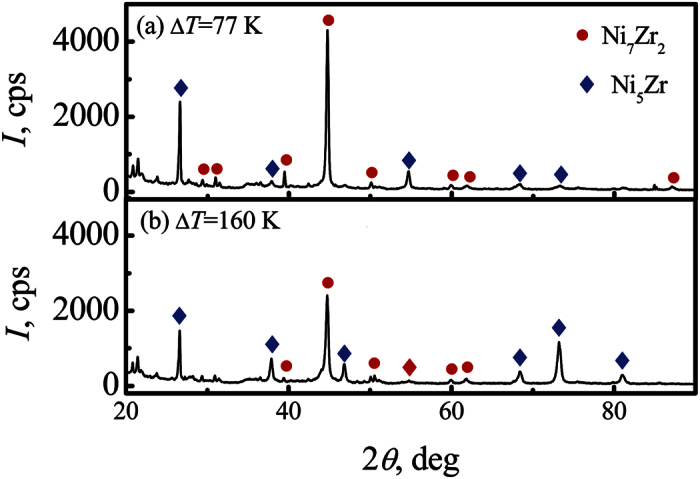
XRD patterns of samples solidified at undercoolings of (**a**) Δ*T* = 77 K and (**b**) Δ*T* = 160 K to reveal the volume change of the two phases.

To further confirm the variation of phase constitution, X-ray diffraction (XRD) patterns of samples solidified at two different undercoolings of 77 K and 160 K are shown in Fig. 4. It can be seen that the main peaks of Ni_7_Zr_2_ phase decrease sharply with the increasing undercooling. On the contrary, the peaks of Ni_5_Zr phase increase with the enhancement of undercooling. This indicates that the volume fraction of Ni_5_Zr phase at high undercooling is larger than that at low undercooling.

According to the above microstructures presented in Fig. 3, two growth modes can be concluded. If the undercooling is smaller than the critical value of Δ*T*_crit_ = 124 K, the Ni_7_Zr_2_ phase is preferred to primarily nucleate and grow into the manner of dendrites during the rapid solidification of the undercooled melt, which results in a sudden temperature rise due to the released heat of crystallization. Subsequently, with the decrease of temperature, the peritectic phase Ni_5_Zr starts to nucleate at the surface of the Ni_7_Zr_2_ dendrites when the temperature drops below the peritectic temperature *T*_P_. In this case, the primary Ni_7_Zr_2_ dendrites, peritectic phase Ni_5_Zr and liquid phase will contact with each other at a triple junction, which is the requirement of peritectic reaction. Then, peritectic reaction of Ni_7_Zr_2_ + L → Ni_5_Zr takes place and peritectic phase Ni_5_Zr grows along the surface of Ni_7_Zr_2_ dendrites to form a thin peritectic layer. Peritectic reaction, which is governed by local short range diffusion of the solute in the melt ahead of the primary Ni_7_Zr_2_ dendrites and peritectic phase Ni_5_Zr, can proceed rapidly at the initial stage of the peritectic growth process. Whereas, once the primary Ni_7_Zr_2_ dendrites are enwrapped by peritectic phase Ni_5_Zr, peritectic phase Ni_5_Zr will separate the primary phase Ni_7_Zr_2_ and liquid phase, leading to the disappearance of the triple junction and the cease of peritectic reaction. After which, the peritectic phase Ni_5_Zr grows into the primary Ni_7_Zr_2_ dendrites by peritectic transformation. Since the peritectic transformation is controlled by long range solid-state diffusion, it proceeds sluggishly. Unfortunately, the cooling rate in the experiments is about 15 K/s, which results in that peritectic transformation could not proceed completely^11^ and only a small amount of primary dendrites could transform to peritectic phase by peritectic transformation. Therefore, the primary phase is always retained in the final microstructures after peritectic solidification, as shown in Fig. 3(a–c). Due to the existence of the Ni_7_Zr_2_ phase, the composition of residual liquid deviates from the initial composition of the melt and moves to eutectic zone according to the phase diagram in Fig. 2(c). Hence, the residual liquid solidifies as eutectic when the temperature drops below the eutectic temperature, which is presented in Fig. 3(b).

Once the undercooling exceeds the critical value of Δ*T*_crit_ = 124 K, there exists only a small amount of Ni_7_Zr_2_ phase in the solidified microstructure, as illustrated in Fig. 3(d). There are two possible solidification paths for the formation of such a microstructure. The first possibility is that only a small amount of Ni_7_Zr_2_ phase forms first but peritectic phase Ni_5_Zr prefers to grow from the undercooled melts. Thus, the microstructure is composed of predominant peritectic phase Ni_5_Zr and a small amount of Ni_7_Zr_2_ phase. The second possibility is that Ni_5_Zr phase primarily grows in the undercooled melt. Since the atoms of different species have to sort themselves onto proper lattice place during the growth of intermetallic compounds, the growth velocity of Ni_5_Zr phase is sluggish. The released heat of crystallization results in a steep rise of temperature and a decrease of interface undercooling. Thus, the growth of peritectic Ni_5_Zr phase cannot proceed to completion and a small amount of liquid remains in the inter-dendritic region. The undercooling of residual liquid after recalescence is smaller than 124 K according to the temperature data in Fig. 2(b). In this case, Ni_7_Zr_2_ is preferred to grow from the residual undercooled liquid. Actually, due to less released heat and high cooling rate during the solidification of Ni_7_Zr_2_ phase, the second recalescence is difficult to distinguish from the undulations in the pyrometer signal in Fig. 2(b). The second possibility is more likely to occur according to our previous study^3^. We suggest that peritectic phase Ni_5_Zr preferentially grows when the undercooling is larger than 124 K. It is verified again that the equilibrium solidification of Ni_7_Zr_2_ phase is replaced by the direct growth of peritectic phase Ni_5_Zr when the undercooling is beyond the critical value of 124 K.

**Figure 5:**
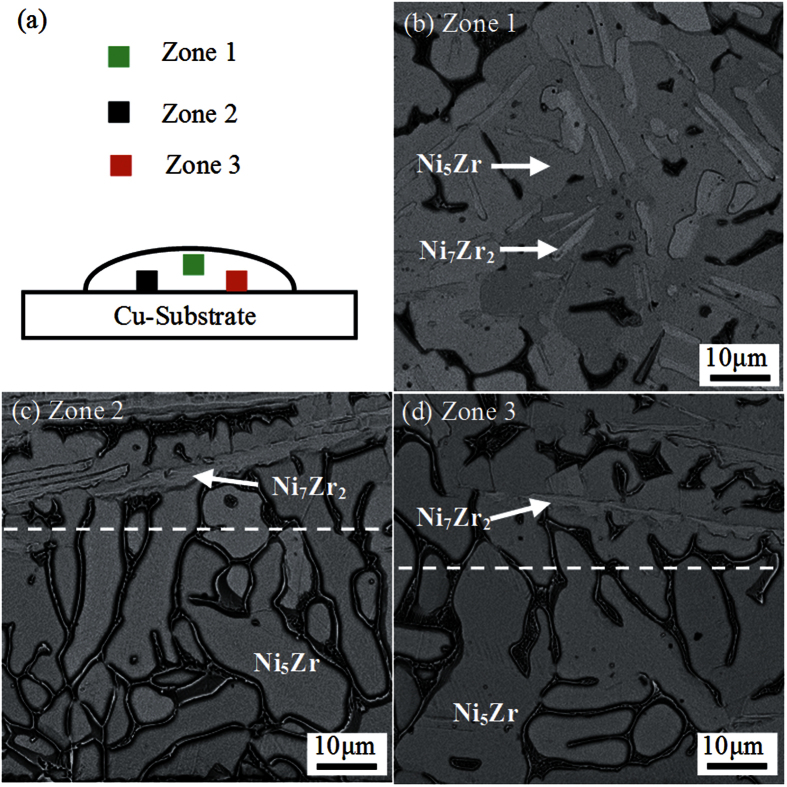
Cross-sectional SEM image of the sample dropped onto the Cu-substrate after undercooled to 160 K. (**a**) Schematic of the Cross-sectional view of the dropped sample. (**b**) Microstructure in Zone 1. (**b**) Microstructure in Zone 2. (3) Microstructure in Zone 3.

A heat flux from the melt to the surrounding is necessary during the solidification, which is dominated by cooling rate. If the cooling rate of undercooled Ni_83.25_Zr_16.75_ melt is sufficiently high, the crystallization heat would be rapidly transferred to the surrounding during the growth of peritectic phase Ni_5_Zr. This will result in the continuous growth of peritectic phase Ni_5_Zr and the formation of phase-pure peritectic phase Ni_5_Zr microstructure. In the case of electromagnetic levitation, heat is mainly transferred by flowing helium gas. To obtain high cooling rate and verify the speculation, a Ni_83.25_Zr_16.75_ sample was undercooled up to 160 K and then quenched on a Cu-substrate. The cross-sectional micrographs of different zones in the quenched sample are shown in Fig. 5. Figure 5(b) presents the microstructure away from the Cu-substrate, which consists of Ni_7_Zr_2_ dendrites, peritectic phase Ni_5_Zr and eutectic. The microstructures on the Cu-substrate side are illustrated in Fig. 5(c,d). Obviously, the microstructure is composed of two regions. The upper one is characterized by the primary Ni_7_Zr_2_ dendrites enveloped by peritectic phase Ni_5_Zr. The below one adjacent to the Cu-substrate consists of only peritectic phase Ni_5_Zr with no primary phase Ni_7_Zr_2_ because high cooling rate is obtained at the interface between the Cu-substrate and melt. To check the reproducibility of the observation, the quench experiments were performed on two samples and the results agree well. This suggests that peritectic phase Ni_5_Zr directly solidifies by completely suppressing the growth of the primary Ni_7_Zr_2_ dendrites. Furthermore, this is an evident proof for the transition of the primary growth mode from Ni_7_Zr_2_ phase to peritectic Ni_5_Zr phase when the undercooling exceeds the critical value of 124 K.

Dendritic growth is the major growth mode in undercooled melts, which determines the evolution of the solidified microstructure. Meanwhile, dendritic growth is controlled by the temperature and concentration gradients, resulting from the heat and solute transport around the solid-liquid interface. To analyze the dendrites growth kinetics of Ni_7_Zr_2_ and Ni_5_Zr, a LKT/BCT model^19^,^20^,^21^ is adopted to describe the dendritic growth as a function of undercooling. The physical parameters used in the calculations are obtained by molecular dynamics simulation and linearly fitting the values of pure metals^22^, which are listed in Table 1. The calculated dendritic growth velocities of Ni_7_Zr_2_ and Ni_5_Zr phase are shown in Fig. 2(a). Evidently, the calculated results of Ni_7_Zr_2_ phase are in good agreement with the experimental results when the undercooling is smaller than 80 K. The dendritic growth mechanism of Ni_7_Zr_2_ phase is mainly governed by solute diffusion. Similarly, the calculated dendritic growth velocity of Ni_5_Zr phase is also close to the experimental values. The initial composition of the melts is the same as that of peritectic phase Ni_5_Zr. Hence, if peritectic phase Ni_5_Zr preferentially grows, mass transport by segregation and constitutional effects can be neglected^23^, thus, the constitutional undercooling Δ*T*_c_ = 0. The curvature undercooling is usually small due to the large curvature radius of dendrites, which also can be neglected. Therefore, the dendritic growth of Ni_5_Zr phase is controlled by thermal undercooling and kinetic undercooling. In other words, thermal diffusion and liquid-solid interface atomic attachment kinetics play a vital role in determining the growth velocity of Ni_5_Zr phase.

## Conclusion

In summary, the dendritic growth in undercooled Ni_83.25_Zr_16.75_ peritectic alloy was investigated by electromagnetic levitation method. The maximum undercooling achieved in the experiment is 198 K. The dendritic growth velocity shows a steep acceleration around a critical undercooling of Δ*T*_crit_ = 124 K, which gives the evidence of the transition of the primary growth mode from Ni_7_Zr_2_ phase to Ni_5_Zr phase. This is ascertained by combining the temperature-time profile and the evolution of the microstructures. The solidified microstructure is composed of coarse Ni_7_Zr_2_ dendrites, peritectic phase Ni_5_Zr and eutectic structure when the undercooling is less than the critical undercooling of Δ*T*_crit_ = 124 K. However, only a small amount of Ni_7_Zr_2_ phase appears in the solidified microstructure once the undercooling exceeds the critical value of 124 K, which indicates that the peritectic phase Ni_5_Zr primarily solidifies. Furthermore, In the case of dropping the undercooled sample of 160 K onto a Cu-substrate, the microstructure of the quenched sample adjacent to the Cu-substrate consists of only peritectic phase Ni_5_Zr with no primary phase Ni_7_Zr_2_, which suggests that peritectic phase Ni_5_Zr directly solidifies by completely suppressing the growth of the primary phase Ni_7_Zr_2_. The dendritic growth mechanism of Ni_7_Zr_2_ phase is mainly governed by solute diffusion. However, thermal diffusion and liquid-solid interface atomic attachment kinetics play a vital role in determining the growth velocity of Ni_5_Zr phase.

## Experimental Details

Master alloys of Ni_83.25_Zr_16.75_ peritectic alloy were prepared by 99.99% pure Ni and 99.9% pure Zr mixtures in an arc melting furnace. The samples of about 0.6 g were levitated and melted by an electromagnetic levitation facility, which was evacuated to 10^−5^ Pa and backfilled with argon gas to 1 atm. The sample was cooled with flowing helium gas to achieve substantial undercooling. Its temperature was measured using a one-color Raytek Marathon MR1SCSF infrared pyrometer, which was calibrated by a PtRh30-PtRh6 thermocouple. The dendritic growth velocity was determined from the recalescence time measured by a photoelectric detector. The solidified samples and phase constitution were analyzed by a Phenom Pro SEM and a Rigaku D/max 2500 X-ray diffractometer (XRD).

## Additional Information

**How to cite this article**: Lü, P. *et al*. Evidence for the transition from primary to peritectic phase growth during solidification of undercooled Ni-Zr alloy levitated by electromagnetic field. *Sci. Rep.* **6**, 39042; doi: 10.1038/srep39042 (2016).

**Publisher's note:** Springer Nature remains neutral with regard to jurisdictional claims in published maps and institutional affiliations.

## Figures and Tables

**Table 1:** Physical parameters of Ni_83.25_Zr_16.75_ peritectic alloy^22^.

Parameter	Unit	Ni_7_Zr_2_	Ni_5_Zr
Heat of fusion	Δ*H* (J/mol)	21000	18500
Heat capacity	*C*_p_ (J/mol)	39.4	39.8
Liquidus slope	m_l_ (K/at.%)	8.6	0
Melt diffusion coefficient	*D*_L_ (m^2^/s)	1.22 × 10^−7^exp(−54951/*RT*)	1.22 × 10^−7^exp(−54951/*RT*)
Gibbs-Thomosion parameter	*Γ* (mK)	1.40 × 10^−7^	1.41 × 10^−7^
Velocity of sound	*V*_0_ (m/s)	3000	3000
